# Quantitative Proteomics Identifies Potential Molecular Adaptations in Mouse Models of Congenital Stationary Night Blindness Type 2

**DOI:** 10.1016/j.mcpro.2025.101462

**Published:** 2025-11-10

**Authors:** Matthias Ganglberger, Lucia Zanetti, Anna-Sophia Egger, Alexander Günter, Bettina Wagner, Soumaya Belhadj, Regine Mühlfriedel, Dagmar Knoflach, Emilio Casanova, Thomas Rülicke, Mathias W. Seeliger, Marcel Kwiatkowski, Hartwig Seitter, Alexandra Koschak

**Affiliations:** 1 Institute of Pharmacy, Pharmacology and Toxicology Unit, University of Innsbruck, Innsbruck, Austria; 2 Department of Biochemistry and Center for Molecular Biosciences Innsbruck, Austria; 3 Division of Ocular Neurodegeneration, Institute for Ophthalmic Research, University of Tübingen, Tübingen, Germany; 4 Department of Biomedical Sciences, University of Veterinary Medicine, Vienna, Austria; 5 Institute of Pharmacology, Center of Physiology and Pharmacology & Comprehensive Cancer Center (CCC), Medical University of Vienna, Vienna, Austria; 6 Ludwig Boltzmann Institute for Hematology and Oncology, Medical University of Vienna, Vienna, Austria

## Abstract

Pathogenic variants in the *CACNA1F* gene are linked to congenital stationary night blindness type 2 though their specific molecular effects remain elusive. This study examines the retinal impact of two variants: a truncation (RX) and a gain-of-function (IT) to explore variant-specific retinal proteome changes. Electroretinography showed that RX primarily affects rod pathways, while IT disrupts both rod and cone signaling, consistent with morphological findings. Comprehensive quantitative proteomic analysis using mass spectrometry identified approximately 4000 proteins across wild-type control and mutant retinas, including also low-abundant membrane proteins. IT retinas exhibited widespread proteomic remodeling suggesting broad cellular responses and also compensatory molecular adaptations. In contrast, RX retinas displayed a more restricted profile. Similar to IT retinas, we found reduced Cav1.4 protein levels but without transcriptional downregulation in RX, alongside selective changes in synaptic proteins such as Erc1, Lrfn2, vGlut1, and Rab3a. These findings suggest selective molecular changes in synaptic organization and calcium-related pathways in RX retinas, offering insights into the mechanisms of Cav1.4 dysfunction in retinal disease. Deep proteomic analysis demonstrates how retinal cells reorganize their molecular architecture in response to calcium channel defects and highlights the utility of comprehensive proteomics to characterize adaptive cellular responses to genetic perturbations in retinal synaptic organization.

## Highlights

• RX causes a milder retinal phenotype compared to IT, with less remodeling and preserved outer nuclear layer integrity.
• RX primarily affects rod pathways, while IT disrupts both rod and cone signaling.
• Novel retinal synapse enrichment protocol successfully enables detection of Cav1 channel protein.
• Proteomic changes occur in both, with IT showing greater protein dysregulation than RX.
• RX retinas show reduced Cav1.4 levels and selective synaptic changes, including Erc1 and Lrfn2.

## Graphical Abstract

**Figure:**
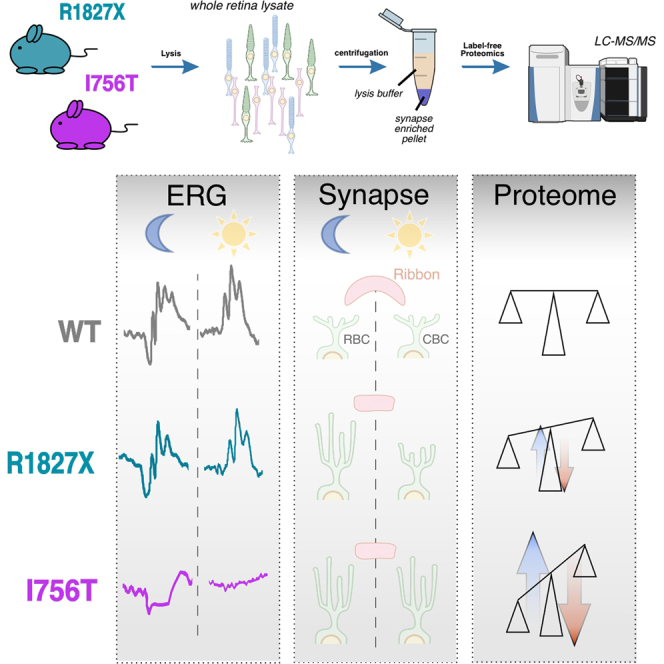

## In Brief

Pathogenic variants in the CACNA1f gene cause congenital stationary night blindness type 2, yet their molecular effects remain unclear. This study compares two variants: a truncation (RX) and a gain-of-function (IT). Proteomic analysis identified ∼4000 proteins, revealing widespread dysregulation in IT retinas and synaptic changes in RX retinas. RX primarily affects rod pathways, while IT disrupts both rod and cone signaling. These findings highlight how proteomics uncovers adaptive molecular responses to calcium channel dysfunction in retinal disease.

**Fig. 1:**
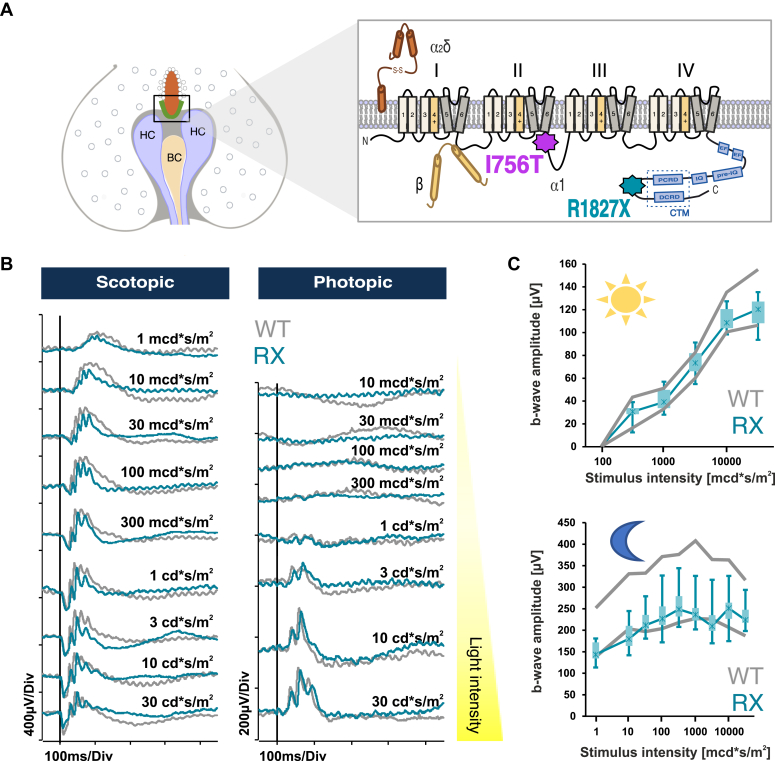
**Localization of the CSNB2 variants and functional characterization of RX retinas.***A*, localization of the Arg1827X and I756T variant. *B*, *in vivo* retinal functionality was assessed using single flash full-field ERGs series in WT and RX mice under both dark- and light-adapted conditions. The *vertical line* indicates the onset of the flash stimulus. ERG recordings were only performed for RX mice, as the ERG phenotype of IT mice has been reported previously (6). *C*, quantitative evaluation of the b-wave amplitudes (Box-and-Whisker plot) of the photopic and scotopic ERG series from WT: N = 5 and RX: N = 6. *Boxes*: 25 to 75% quantile range, *whiskers*: 5% and 95% quantiles, *asterisks*: median. CSNB2, congenital stationary night blindness type 2; ERG, electroretinography.

Congenital stationary night blindness type 2 (CSNB2, OMIM 300110) represents a compelling paradox: while caused by mutations in a single gene, the clinical manifestations are clearly variable between patients. This X-linked condition results from pathogenic variants in the *CACNA1*F gene encoding Cav1.4 (α1F) L-type calcium channels, which are essential for precise calcium regulation in retinal neurons and enable tonic glutamate release at photoreceptor and bipolar cell synaptic terminals (1, 2). The resulting dysfunction impairs transmission between photoreceptors and second-order neurons, manifesting as diverse visual symptoms including reduced visual acuity, strabismus, nystagmus, and photophobia, though night blindness may not always be present (2, 3, 4, 5). Over 140 Cav1.4 variants have been documented (2, 5) which can produce distinct functional consequences that might also translate to variable clinical severity. Although two variants cannot capture this entire spectrum of disease impact, we chose two following contrasting variants to demonstrate how proteomics can reveal variant-specific molecular consequences. The I745T substitution (I756T in mice, corresponding to reference sequence Q9JIS7; IT in the following, Fig. 1*A*) represents the severe end, causing a strong hyperpolarizing shift in channel activation (∼−30 mV) (7) and producing unusually severe symptoms in humans that affect not only hemizygous males but also heterozygous female carriers as well as their children, indicating an early onset of the condition (8). In contrast, the R1816X truncation variant (R1827X in mice, corresponding to reference sequence Q9JIS7, RX in the following, Fig. 1*A*) produces a milder phenotype, with patients experiencing transient photophobia that resolves with age instead of persistent night blindness (9).

Mouse models have provided crucial insights into how these variants differentially impact retinal structure and function. The IT variant drives progressive retinal degeneration characterized by immature ribbon synapse morphology, extensive cellular sprouting, and ultimately severe photoreceptor loss by 8 months of age (6, 10, 11, 12, 13). Electroretinography (ERG) revealed reduced b-wave responses under both scotopic and photopic conditions, with additional a-wave reductions suggesting photoreceptor degeneration (12). The RX variant, while displaying altered calcium-dependent inactivation properties (14), presented a markedly different trajectory whose *in vivo* consequences were so far poorly understood.

Understanding how cells adapt to different variant types could provide insights that traditional approaches that focus either on individual proteins or specific cellular pathways might miss. We therefore employed an unbiased, label-free quantitative proteomic approach to map the global retinal proteome landscape. Our proteomic strategy offers the advantage to capture comprehensive protein level changes that occur in response to different variants, potentially revealing unexpected molecular mechanisms.

By comparing proteomic profiles between IT and RX mouse models, we aimed to uncover how different *CACNA1F* variants influence retinal responses. In this study, we first compare the retinal phenotype of the novel RX variant with the well-established IT variant, which is associated with a more severe clinical presentation. We then employ a comprehensive proteomic analysis to investigate molecular changes in the retina, focusing on synaptic and calcium-handling pathways.

## Experimental Procedures

### Experimental Design and Statistical Rationale

We established a protocol to enrich synaptic proteins from the retina using a two-step centrifugation workflow after cell lysis. Proteins were analyzed in data-independent acquisition (DIA) mode. After enrichment, protein concentrations were measured using the bicinchoninic acid assay. Membrane - enriched retinal lysates were separated on a gradient gel, followed by in-gel digestion with trypsin and subsequent liquid chromatography tandem mass spectrometry (LC-MS/MS) analysis. Protein groups and their normalized intensities were quantified using DIA-NN software and used for downstream analysis.

Each replicate consisted of the two retinas pooled from three animals. We included three replicates per genotype: RX, IT, and wild-type control. Detailed statistical and bioinformatic methods are described in the “Proteome Analysis by LC-MS/MS” and “Bioinformatics Data Processing of Proteome LC-MS/MS Data” sections.

### Mice

Animals were housed in groups of 2 to 5 per cage (type II-long, size: 20 × 37 × 15 cm) under standard laboratory conditions (12:12 light/dark, lights on at 07:00 h, 22 ± 2 °C, 50–60% humidity) with food and water available ad libitum. Experimental procedures were designed to minimize animal suffering and the number of used animals and approved by the national ethical committee on animal care and use (Austrian Federal Ministry for Science and Research). All methods were performed in accordance with the relevant guidelines and regulations.

**Fig. 2:**
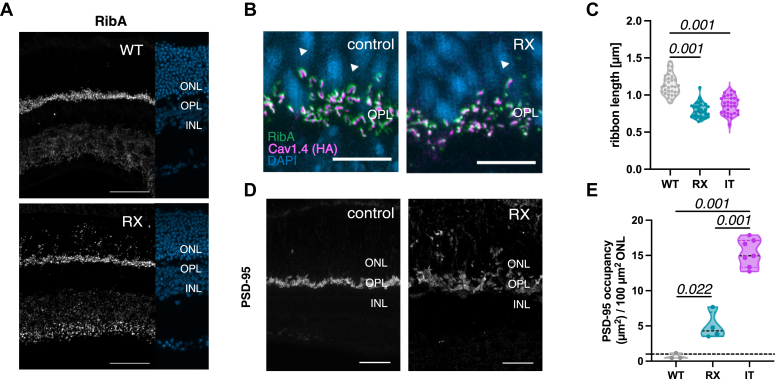
**Photoreceptor synapse structure and protein composition.***A*, *left*: staining of ribeye A, the main protein of the ribbon synapse was labeled with ribeye A; *right*: DAPI staining; WT: N = 5, RX: N = 6. The scale bar represents 50 μm. *B*, colocalization of Cav1.4 channels (*magenta*, anti-HA) and ribeye A (*green*) remain in OPL marked with a triangle. control: N = 4, RX: N = 3. The scale bar represents 10 μm. *C*, measurement of the ribbon length: WT: 1.13 μm ± 0.025, N = 4, n = 31; RX: 0.79 μm ± 0.018, N = 4, n = 31; IT: 0.85 μm ± 0.022, N = 4, n = 30. *D*, staining of anti-PSD-95; WT: N = 4; RX: N = 5. The scale bar represents 20 μm. *E*, photoreceptor terminal occupancy in the ONL comparing wild type (WT: 0.68% ± 0.24, N = 3) and RX retinas (RX: 4.96% ± 0.95, N = 4), as well as previously published results for Cav1.4-IT (IT: 15.35% ± 0.74, N = 7; (10)). Statistical analysis: one-way Anova with Tukey post hoc test; ns: *p* > 0.05; *p* value is indicated above; only significant *p* values are stated. ONL, outer nuclear layer, OPL, outer plexiform layer, INL, inner nuclear layer; DAPI, 4′,6-diamidino-2-phenylindole.

To generate the RX (*B6.-Cacna1f*^*tmR1827X(Biat)*^*/Kos*) mouse strain, the targeting vector was designed to produce a conditional knock-in mouse model carrying the Arg1827X *CACNA1F* (Fig. 1*A* and Supplemental Fig. S1*A*) with hemagglutinin (HA) and FLAG tags at the C terminus for immunohistochemical detection of the channel. The final construct was inserted in the pBlue Script II SK (+) vector with PCR fragments spanning the Cacna1f genomic region starting from exon 43 to 48, flanked by a loxP site. To sustain the reading frame the constructs start with a semi-intron. To generate gene targeted embryonic stem (ES) cells 1 × 107 (1 × 107) C2 ES cells (Parental ES cell line C57BL/6NTac, Stock Number: 011989-MU, Citation ID: RRID: MMRRC_011989-MU, A. Nagy Basic ES Cell line) were electroporated with 15 μg of linearized targeting vector. Neomycin resistance was used to select for targeted ES cell clones. Resistant clones were screened for correct integration of the targeting construct by PCR and archived. The presence of the targeted allele was confirmed by Southern blot analysis. To generate genetically modified mice host blastocysts were produced by superovulation of BALB/cJRj females and injected with correctly targeted ES cells. High-percentage male chimeras (>80%) were bred with C57BL/6NRj females and the offspring were selected by coat color and genotyped by PCR (forward primer 5′-GGAATAGAGGGTAACACAAGAAG, reverse primer 5′-ACTCACTACATAGACCAGGC) to distinguish between wildtype and knock in mice. Generation of gene targeted mice was performed in accordance with the Austrian Animal Experiment Act (animal experiment license number BMBWF-68.205/0010-V/3b/2019). (Supplemental Fig. S1*B*). Subsequent crossbreeding with a CAG-Flp mouse generated the wildtype mouse line carrying a HA-tag at the end of the C terminus to detect the channel immunohistochemically. Afterward, these mice were crossbreed with CMV-Cre (B6.C-Tg(CMV-cre)1Cgn/J) mouse, was used to generate the RX mouse strain. Mouse ear punches were used for genotyping. Mice were backcrossed to the C57BL/6J background for at least five generations. Since the *Cacna1f* gene encoding Cav1.4 is on the X chromosome, only males of each genotype were used for experiments. Littermates were used as controls, except for Figure 2*B* were we used the HA tagged wild type mice. IT mice were described in (6).

### Immunohistochemistry

Six- to ten week-old mice were killed by cervical dislocation. Vertical sections: the following steps were conducted at room temperature if not stated otherwise. Eyes were quickly removed from the eye socket, opened at the scleral-corneal rim. Cornea, lens, and vitreous were removed and eyecups were washed three times in phosphate-buffered saline (1X PBS, pH 7.4). Subsequently, the eyes were fixed with 4% paraformaldehyde in 1X phosphate-buffered saline (1X PBS, pH 7.4) for 20 min. Afterward, washed four times with 1X PBS and cryoprotected by a graded sucrose series: 10% sucrose in 1X PBS and 20% sucrose in 1X PBS for 1 hour each and 30% sucrose in 1X PBS overnight at 4 °C. Eyecups were orientated along the dorsoventral axis, embedded in OCT Medium (Tissue-Tek O.C.T Compound; Sakura Finetek) and frozen in liquid nitrogen. Vertical sections (14 μm) were cut on a cryostat (Leica Microsystems), mounted on gelatine coated slides and stored at −20 °C. For immunofluorescence experiments, sections were washed three times in 1X PBS-T (1X PBS + 0.1% Triton X-100, Sigma-Aldrich), blocked for 1 hour in 1X PBS-T containing 1% bovine serum albumin (Sigma-Aldrich, A7030) and incubated overnight at 4 °C with primary antibodies diluted in 1X PBS-T at concentrations listed in Table 1. After washing three times with 1X PBS-T, sections were incubated with the secondary antibodies (Table 2) for 1 hour. Additional washes preceded the counterstaining with 4′,6-diamidino-2-phenylindole) (1:10,000; Sigma-Aldrich, D-9542) and subsequently mounted using Poly/Mount (Polysciences, Inc).

**Table 1:** Primary antibodies

Primary antibody	Dilution	Company	Order number
Anti-PKCα (rabbit)	1:500	Santa Cruz Biotechnology	Sc208
Calbindin (rabbit)	1:1000	Swant	CB-38a
Secretagogin (sheep)	1:1000	BioVendor	RD184120100
PSD-95 (rabbit)	1:1000	Synaptic Systems	124002
Ribeye (rabbit)	1:1000	Synaptic Systems	192103
Anti-HA (rat)	1:1000	Roche	ROAHAHA
Iba1 (rabbit)	1:750	GeneTex	GTX1000042
NF 200 (chicken)	1:1000	Biolegend	822601
Anti-Cav1.4 (rabbit)	1:1000	Synaptic systems	365003
Sodium potassium ATPase (rabbit)	1:100.000	Abcam	Ab76020

**Table 2:** Secondary antibodies

Secondary antibody for IHC	Dilution	Company	Order number
Alexa Fluor 568 donkey-anti-sheep IgG	1:400	Abcam	Ab175712
Dylight 650 donkey-anti-mouse IgG	1:400	Invitrogen	10169
Alexa Fluor 488 goat-anti-mouse IgG	1:400	Invitrogen	A11001
Alexa Fluor 568 goat-anti-mouse IgG	1:400	Invitrogen	A11004
Alexa Fluor 488 goat-anti-rat IgG	1:400	Invitrogen	A11006
Alexa Fluor 568 goat-anti-rabbit IgG	1:400	Invitrogen	A11011
Alexa Fluor 488 goat-anti-chicken IgG	1:400	Invitrogen	A11039

Secondary antibody for WB	Dilution	Company	Order Number
Anti-rabbit IgG peroxidase antibody	1:15.000	Sigma Aldrich	A0545

IgG, immunoglobulin G; WB, western blot; IHC, immunohistochemistry.

Antigen retrieval was done for unmasking the HA epitope to detect Cav1.4 signals in retinal slices (Fig. 2*B*). Sections were incubated with retrieval buffer (Tris Base 10 mM, EDTA 1 mM, Tween 20 0.1%, distilled water, pH 8.9) in a water bath (97 °C) for 2 min.

### Image Acquisition

Sections were imaged with AxioObserver Z1 microscope equipped with an Apotome 40x/1.4 oil objective and an Axiocam 702 camera or a confocal laser scanning microscope (Leica TCS SP5-II; Leica Microsystems) at 40 × magnification (NA 1.30). Series of micrographs were taken at 0.25 and 0.42 μm intervals. Magnification and exposure time were adjusted for each experiment to the condition of the brightest signal. For presentation in figures as well as for quantification, regions of interest were selected and z-stack were collapsed to a z-projection with maximum intensities via the Zen blue software. Brightness and contrast were adjusted for better visibility.

### Occupancy Measurement and Quantification

To measure neurite occupancy of the outer nuclear layer (ONL) by horizontal cells (HCs), rod bipolar cells (RBCs), and cone bipolar cells (CBCs) as well as photoreceptor occupancy, sections were labeled with antibodies against calbindin (HCs), PKCa (RBCs), and secretagogin (CBCs). Z-stacks were collapsed and 8 bit images were subsequently converted into a binary image in imageJ (National Institutes of Health). 4′,6-Diamidino-2-phenylindole stained retinal layers were used as binary masks. Occupancy analysis (ObjectFinder; https://lucadellasantina.github.io/ObjectFinder/) within the ONL area above the outer plexiform layer (OPL) border was performed and was calculated as a function of the total area analyzed in the binary mask. Images used for analyses were collected from central as well as peripheral parts of the retina.

### Electroretinography

The functional implications of the RX variant were assessed *in vivo* via ERG. Binocular ERGs were recorded using gold-wired ring electrodes placed on the corneal surfaces of mice at six postnatal weeks of age as described previously (15). In short, mice were anaesthetized using Ketamine (66.7 mg/kg body weight) and Xylazine (11.7 mg/kg body weight). Their pupils were dilated with tropicamide drops and single flash ERG responses were obtained under scotopic (dark-adapted overnight) and photopic (light-adapted with a background illumination of 30 cd/m^2^ starting 5 min before recording) conditions. Single white-flash stimuli ranged from 1 mcd∗s/m^2^ to 30 cd∗s/m^2^ under dark-adapted and from 10 mcd∗s/m^2^ to 30 cd∗s/m^2^ under light-adapted conditions. Each recording step was averaged 10 to 15 times, with interstimulus intervals of 2 s (1–300 mcd∗s/m^2^), 5 s (1–3 cd∗s/m^2^), or 10 s (10–30 cd∗s/m^2^). Responses to trains of flashes (flicker) were obtained under dark- and light-adapted conditions using a fixed intensity (3 cd∗s/m^2^ for DA and 10 mcd∗s/m^2^ for LA; resembling the International Society for Clinical Electrophysiology of Vision standard flash (ISCEV SF). Responses were averaged 20 times for stimuli between 0.5 to 3 Hz and 30 times for frequencies above 5 Hz. A band-pass filter cutoff frequency of 0.3 and 300 Hz was applied. The flash duration was 4 ms for all ERG recording protocols. ERGs were obtained in RX (N = 6) and wild type control animals (N = 5). Full-field ERG recordings were performed with the Espion E3 console connected to a computer, a 32 bit amplifier and a Ganzfeld Bowl (Diagnosys, LLC). ERG performed in this study adhered to the ARVO Statement for the Use of Animals in Ophthalmic and Vision Research and was approved by the competent legal authority (Regierungspräsidium Tübingen). All procedures involving animals were conducted in accordance with the German Animal Welfare Act. The noninvasive retinal imaging in genetically defined mouse strains was notified to the relevant authorities under Section 8a (1) No. 2 of the German Animal Welfare Act (TierSchG) and submitted on March 12, 2018.

### Microelectrode Array Recordings

Mice were dark-adapted for at least 2 h before the experiment and sacrificed by cervical dislocation after isoflurane anesthesia between circadian zeitgeber time ZT5.5 and ZT8.5. Animals were 6 to 8 weeks old at the time of the experiment. Before enucleation, the ventral position on each eye was marked with marker pen and the eyes were placed in bath solution (in [mM]: 110 NaCl, 2.5 KCl, 1 CaCl_2_, 1.6 MgCl_2_, 10 D-Glucose, and 22 NaHCO_3_; bubbled with 5% CO2/95% O2) for dissection. After removing cornea and lens, the isolated retina was mounted on a dark gray nitrocellulose filter (13006–50-ACN, Sartorius Stedim) with a 3 x 3 mm aperture ensuring the dorsal part was as centered as possible. All procedures were performed under dim red light.

Recordings were carried out with perforated 120-electrode micro-electrode arrays (MEA; 120pMEA100/30iR-Ti-pr, Multichannel Systems). The dorsal retina was placed GCs-side down in the recording chamber. Dorsal part was chosen based on cone opsin spectral distribution and visual stimulation spectra. The tissue was continuously perfused with fresh bath solution at 30 °C and raw data were recorded at 25 kHz with a MEA-system (MEA2100, Multichannel Systems). Light stimulation was delivered with a computer-controlled digital light processing projector (Lightcrafter E4500MKII, EKB Technologies Ltd) using a grayscale visual stimulus. The projector output was limited to a ±50% Weber contrast range around a mean background (gray value 200 ± 48). Built-in blue and green LEDs were used to match rhodopsin and M-opsin spectra. The light path was integrated with neutral density (ND) filters (63–390, 63–393, 63–395; Edmund Optics) to enable scotopic (ND8) and photopic (ND4) light stimuli. The same set of stimuli was presented at each ND level.

Retinal spikes were extracted from high-pass filtered (500 Hz, 10th-order Butterworth filter) traces using MATLAB (The MathWorks Inc). Spike sorting and analysis followed (16), using a custom MATLAB script.

• Full-field flashes: Consisted of 1-s contrast steps (±50% Weber contrast) with 5-s background gray (gray value 200) between flashes. The maximum positive response after each bright or dark flash was analyzed. Response latency was defined as the time when the smoothed mean firing rate exceeded 37% above background.
• Full-field Gaussian flickering (GF): Consisted of 60 Hz flickering with intensity values drawn from a Gaussian gray value distribution. GF was used to calculate the spike-triggered average and obtain the linear filter of GCs, as described in (17).

### Protein Preparation and Western Blot

Membrane protein preparation from adult mouse retinas (one sample = retinas of three animals pooled) was performed as described previously in (18). In short, tissue was homogenized in ice-cold lysis buffer: 10 mM Tris–HCl (pH 7.4) and protease mix: 0.2 mM PMSF, 0.5 mM benzamidine, 1 M pepstatin, 2 mM iodoacetamide, 1 mg/ml leupeptin, 1 mg/ml aprotinin, and 100 mg/ml trypsin inhibitor. Protein concentration was measured via Bradford assay as per the manufacturer’s protocol. SDS-PAGE and the western blot procedure were used as previously reported (19), except that we used primary and secondary antibodies as indicated in Tables 1 and 2.

### Quantitative real-time PCR

For RNA Isolation the Monarch Total RNA Miniprep kit was used (New England Biolabs) following the manufacturer protocols. Contaminating genomic DNA was removed by DNase I digestion (Qiagen). The isolated RNA concentrations and purities were determined on a Nanodrop spectrophotometer (Thermo Fisher Scientific). Subsequently, complementary DNA (cDNA) synthesis from up to 1 μg of RNA mouse retina was performed using LunaScript RT kits (NEB) containing a mixture of random hexamer and oligo dT primers following manufacturer instructions and the resulting complementary DNAs were stored at −20 °C until use.

Gene expression quantification was performed by probe-based quantitative real-time PCR (qPCR) using off the shelf and custom-made TaqMan assays (Thermo Fisher Scientific) for which standard curves were established as described elsewhere (20) to allow absolute quantification. In brief, qPCR was run on a real-time PCR machine (qTOWER3 Real-Time PCR Thermal Cycler, Analytik Jena) in duplicates in a 96-well format with the following composition per reaction: 10 μl Luna Universal Probe qPCR 2X master mix (NEB), 4 μl water, 1 μl assay, 5 μl cDNA (pre-adjusted to 0.4 ng/μl = 2 ng RNA-equivalent per reaction) or 5 μl water for negative controls, using the standard ramp speed program for probe-based assays. Cycle thresholds (C_T_) were determined at 0.1 ΔRn of the amplification curves and molecule numbers were calculated as described before (6) based on standard curves for each assay. The assays used are summarized in Table 3.

**Table 3:** Assays used for absolute quantification via qPCR

Gene	Exon boundary	Assay ID
Cacna1f	Ex17 – Ex18	Mm00490443_m1
Reference gene		
b2M (beta-2-microglobulin)		Mm00437762_m1

### Gel-Based Proteome Analysis Membrane-Enriched Retina Samples

#### Peptide Preparation for Proteomic Studies

Protein concentration was measured via bicinchoninic acid assay as per the manufacturer’s protocol. Membrane-enriched retinal lysates (20 μg; one sample = retinas of three animals (n = 9 mice per condition), described in “Protein preparation and western blot) were loaded on a gradient gel 3 to 8% tris-acetate gel and run for 40 min at 50 V. Subsequently, gel was stained with Coomassie dye and the lanes were chopped to approximately 1 mm^3^ pieces and in-gel digested with trypsin followed by mass spectrometry (21).

#### Proteome Analysis by LC-MS/MS

For LC-MS/MS analysis the dried tryptic peptides were reconstructed in 20 μl 0.1% formic acid (FA). The samples were injected into a Vanquish Neo nano ultra-performance liquid chromatography system (nanoUHPLC, Thermo Fisher Scientific) coupled to an Orbitrap eclipse mass spectrometer (Exploris 480, Thermo Fisher Scientific).

The samples were loaded (300 μl/min) on a separation column (DNV PepMap Neo column (75 μm × 500 mm) from Thermo Fisher Scientific (buffer A: 0.1% FA in HPLC-H2O; buffer B: 0.1% FA in acetonitrile) and separated with a gradient of 1% B for 1 min and then increase to 37.5% B in 82 min, increase to 62.5% B in 10 min, increase to 99% B in 0.1 min, keep on 99% B for 2 min, decrease to 1% B in 0.1 min, automatic equilibration. The spray was generated with a heated ESI source.

MS measurements were carried out in DIA. MS1 scan was performed over an m/z range from 400 to 1008, with a resolution of 120,000 at m/z 200 (AGC target = 300%). MS2 acquisition covered the same 400 to 1008 m/z range using 6 m/z isolation windows with a 1 m/z overlap between adjacent windows. MS/MS (MS2 scans) spectra were recorded with a resolution of 15,000 at m/z 200 (normalized AGC target = 3000%, HCD collision energy = 30%).

### Bioinformatics Data Processing of Proteome LC-MS/MS Data

LC-MS/MS raw data were processed and quantified with DIA-NN via library free search (version 1.9.2, (22)). An in silico spectral library was generated from the provided FASTA file (SwissProt, 17,117 entries, downloaded 04.11.2022) using deep learning-based spectral prediction.

The analysis used the following settings: tryptic digestion with cleavage after K/R residues, allowing up to one missed cleavage; peptide length was restricted to 7 to 30 amino acids; precursor m/z range was set to 400 to 1008, with precursor charge states between +1 and + 4. Fragment ion m/z range was set between 200 and 1800. Carbamidomethylation of cysteine was set as a fixed modification, and N-terminal methionine excision was enabled. DIA-NN performed a two-step workflow in which the spectral library was first generated from the DIA runs, and the data were then reanalyzed using this library. Peptides and proteins were identified with a false discovery rate of 1%. Proteins were quantified with the DIA-NN algorithm (using DIA-NN’s default q-value settings, as applied in QuantUMS quantification mode) considering unique peptides.

The post processing of the data was performed in R (version 4.3.3) and RStudio (version 2024.04.2 + 764). Statistical analysis to compare total quantification of identified protein groups was done by two-sided t-tests using rstatix package (https://cran.r-project.org/web/packages/rstatix/index.html). Eulerr package (https://cran.r-project.org/web/packages/eulerr/index.html) was used to generate Venn diagrams. To evaluate the reproducibility of protein intensity measurements across experimental groups, the coefficient of variation (CV) was calculated for each protein as the ratio of the standard deviation to the mean intensity, expressed as a percentage. Protein intensities were grouped by protein IDs and mouse lines, and the mean and standard deviation of CV percentages were computed for each group. A violin plot with overlaid boxplots was generated to visualize the distribution of CVs, with group-specific mean and standard deviation values annotated. Intensity values were log2-transformed and normalized to the median for each condition (Supplemental Fig. S2*C*). For principal component analysis (PCA), the normalized protein group intensities of the proteins reproducibly quantified in all samples were used as an input. For volcano plots, two-tailed *t* tests were performed for all protein groups and adjusted *p* values were calculated using the Benjamini–Hochberg procedure using the rstatix package (https://cran.r-project.org/web/packages/rstatix/index.html). Gene ontology (GO) enrichment analysis was performed using the gprofiler2 package (https://cran.r-project.org/web/packages/gprofiler2/index.html). The rich factor was defined as the ratio of input proteins that are annotated in a term to all proteins that are annotated in this term. The fold enrichment is defined as the ratio of the frequency of input proteins annotated in a term to the frequency of all genes annotated to that term, calculated by dividing geneRatio/BgRatio.

For clustering of the GO results following keywords were used:

• “calcium = c("calcium");
• "synapse" = c("synapse", "synaptic", "transmission", "dendritic", "dendrite");
• "vision" = c("visual", "visible", "phototransduction", "sensory", "light");
• "RNA" = c("RNA", "mRNA");
• "metabolism" = c("metabolic", "biosynthetic", "energy");
• "degeneration" = c("apoptotic", "stress");
• "transport" = c("transport").

To count the protein groups in the respective clusters, we extract the GO pathway with the most proteins associated with.

The r ggplot2 package was used for data visualization (https://cran.r-project.org/web/packages/ggplot2/index.html).

### Statistics

Data presented on graphs were displayed as mean ± SD. Statistical analyses were performed using GraphPad Prism 8 and a *p* value of <0.05 was considered significant. Outliers were identified using ROUT method (Q = 1%) using GraphPad Prism and were excluded from analyses. In qRT-PCR experiments, data were organized and analyzed using MS Excel. N numbers for animals/genotypes as well as the *post hoc* test and corrected *p* values are indicated in the figure legends.

## Results

### Generation and Functional Characterization of a Cav1.4 Mouse Model Mimicking a Human Truncation Variant

To generate the RX mouse model the endogenous Cav1.4 allele was replaced by a C terminally HA and FLAG - tagged variant with a premature stop codon at position 1827 (Supplemental Fig. S1, *A* and *B*). The resulting hemizygous mutants were overall healthy and showed normal sexual activity and reproduction as well as no gross behavioral abnormalities. Cav1.4 protein was detected in retinal lysates of homozygous and heterozygous RX mice by western blot (Supplemental Fig. S1*C*).

To confirm that the RX mouse strain exhibits the typical CSNB2 phenotype, we conducted ERG recordings to assess the functional changes in the retina. Figure 1*B* visualizes a superimposed representative ERG curve recorded at different full-field light flash intensities in RX and wild-type mice under low-light (scotopic, left panel) and bright-light (photopic, right panel) conditions. In both conditions, the a-wave appeared normal in RX retinas, suggesting unaltered electrical activity of presynaptic photoreceptors. However, the b-wave was reduced at scotopic low-light flashes (Fig. 1*B*). Plotting the b-wave amplitude against the light intensities, we found that the mean scotopic b-wave amplitude was at the lower bound of the 95% confidence interval of the wild-type, suggesting a considerable reduction (Fig. 1*C*). In contrast, the photopic b-wave remained was unaffected suggesting that cone-driven responses are preserved. No additional changes in the ERG waveforms were observed, except for a tendency toward delay of the b-wave onset. To provide a more comprehensive analysis, we implemented multielectrode array (MEA) recordings to include the retinal output. Thereby retinal ganglion cell activity was measured by stimulating both rod and cone photoreceptors using full-field light flashes under scotopic and photopic conditions. The ganglion cells were then categorized into two groups: ON and OFF cells based on their responses to dark and bright flashes, respectively. Our analyses revealed that the RX mouse strain exhibited no alterations in the photopic and scotopic OFF pathways. However, we observed a significant delay in the ON response under scotopic conditions, in agreement with the observed tendency in the ERG (Supplemental Fig. S1*D*). This delayed activation of the scotopic ON pathway reflects its primary reliance on rod photoreceptor input (23). Together, our functional data indicate that the RX variant affects only the rod-driven pathway. This contrasts with the IT variant which exhibits a different functional profile affecting both rod and cone pathways (6, 10, 11, 13), consistent with clinical observations in patients (8).

### Effects of Pathogenic Cav1.4 Variants on the Structural Organization of the Photoreceptor Synapse

To evaluate the effects of the RX mutation on photoreceptor structure and synaptic protein organization, we first examined synaptic ribbon formation by labeling Ribeye A protein, a key component of photoreceptor synapses (24, 25). Similar to the IT variant synaptic ribbons were mislocalized in the ONL and exhibited an abnormal punctate morphology (for comparison see (6, 10, 12)), deviating from the characteristic horseshoe-shaped ribbons in wildtype and control mice (wildtype Cav1.4 HA tagged) (Fig. 2, *A* and *B*). However, Cav1.4 (HA tagged) and Ribeye A cooccurred also in the (Fig. 2*B*) indicating that some photoreceptor synapses retain their structure and synaptic connections (Fig. 2*B*), This finding is also consistent with earlier observations reported for the IT variant, as demonstrated in Figure 2*A* (taken from (6) for direct comparison). In addition, we found a significant decrease in ribbon length in both Cav1.4 variants when compared to the wildtype (Fig. 2*C*). These findings corroborate the role of Cav1.4 channels as synaptic organizers (26).

To further examine synaptic integrity, we performed immunostaining for PSD-95, a widely recognized marker for both rod and cone photoreceptor terminals (27). In the CSNB2 retinas, we observed prominent PSD-95 staining in the OPL, similar to that seen in wildtype samples. In addition, we detected ectopic PSD-95 signals in the ONL, which were absent in wildtype retinas (Fig. 2*D*, shown here for RX; see (10) Fig. 6 for IT). However, quantitative analysis showed that the in IT retinas the photoreceptor terminal density in the ONL was approximately three times higher compared to RX retinas (Fig. 2*E*). This finding underscores the stronger impact of the IT variant on photoreceptor terminal structure.

**Fig. 3:**
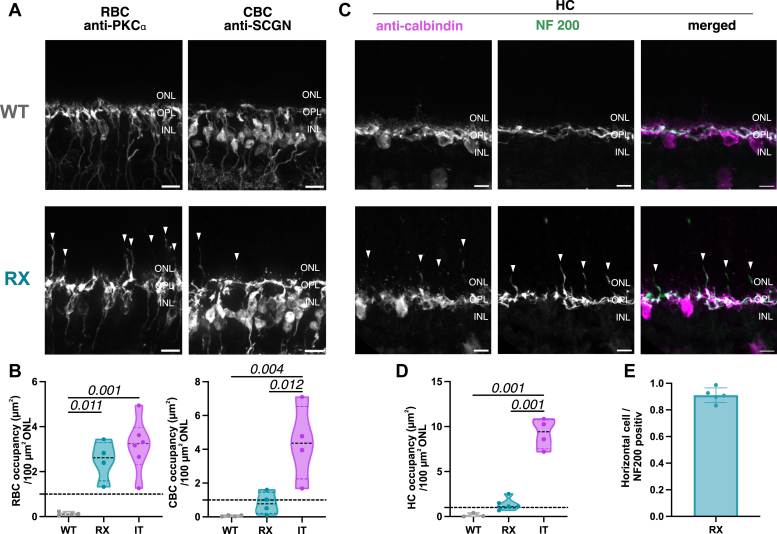
**Immunostaining of second order neurons.***A*, rod bipolar cells (RBCs) were stained with an anti-PKCα antibody in wild type and RX retinas. For cone bipolar cells (CBCs) secretagogin was used as marker. *Arrowheads* indicate elongated bipolar dendrites; (N = 4) and RX (N = 4); the scale bar represents 10 μm. *B*, bipolar cell dendrite occupancy in the ONL among CSNB2 variants. Cone bipolar cell dendrite occupancy in the ONL comparing wild type (0.06% ± 0.02, N = 4) and RX retinas (0.81% ± 0.32, N = 4), as well as previously published results for IT retinas (4.37% ± 0.49, N = 4; (10)). Rod bipolar cell dendrite occupancy: WT (0.13% ± 0.04, N = 4), RX (2.49% ± 0.45, N = 4) and IT retinae (3.16% ± 0.49, N = 6). *C*, double-labeling of horizontal cell (HC) processes with neurofilament 200 (NF200) and anti-calbindin in wild type and RX retinas. *Arrows* indicate axonal sprouting of horizontal cells; WT (N = 3) and RX (N = 5). The scale bar represents 10 μm. *D*, horizontal cell dendrite occupancy: WT (0.15% ± 0.13, N = 3), RX (1.14% ± 0.31, N = 5) and IT retinae (9.22% ± 0.83, N = 4). *E*, ratio of double-labeled horizontal cell dendrites (0.91 ± 0.05, RX: N = 5). Statistical analysis: One-way Anova with Tukey *post hoc* test; ns: *p* > 0.05; *p* value is indicated above; only significant *p* values are stated. CSNB2, congenital stationary night blindness type 2; ONL, outer nuclear layer.

**Fig. 4:**
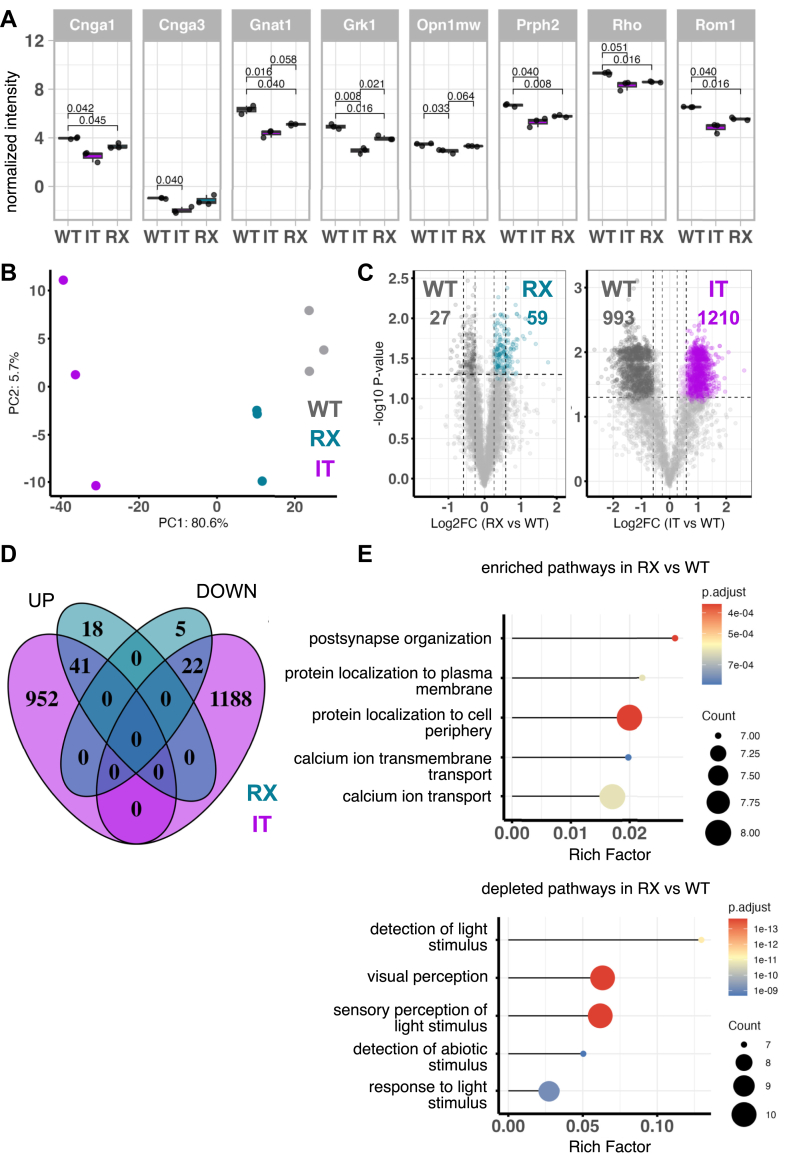
**Proteome analysis of membrane enriched retina samples of CSNB2 variants.***A*, protein abundances of membrane associated photoreceptor marker proteins (Prph2 (P15499), Cnga1 (P29974), Rom1 (P32958), Gnat1 (P20612), Cnga3 (Q9JJZ8), Grk1 (Q9WVL4), Opn1sw (O35599), and Rho (P15409)) were plotted as normalized intensity by median subtraction *(y*-axis) across all mouse models. Statistical analyses with two-tailed unpaired *t* test. N = 9; n = 3 for all pooled samples (WT, RX, and IT) *B*, PCA of normalized protein group intensities from pooled membrane-enriched samples: WT (N = 9, n = 3, *gray*), RX (N = 9, n = 3, *turquoise*), and IT (N = 9, n = 3, *magenta*). *C*, volcano plots depict comparisons of IT and RX versus WT. Significance criteria: adjusted *p* value ≤0.05 (two-tailed unpaired *t* test, Benjamini–Hochberg correction; FDR), with fold change (FC) of 1.2 shown as semitransparent colored dots and FC > 1.5 as solid-colored dots. Dysregulated proteins are colored in the respective color. *D*, Venn diagram display the number of significantly upregulated and downregulated Proteins at FC thresholds of 1.5. *E*, Gene Ontology (GO) enrichment analysis of dysregulated proteins in RX against WT. The dot plot presents the rich factor (*x*-axis) against the number of proteins (*y*-axis), with adjusted *p* values (p.adjust) represented by color coding. CSNB2, congenital stationary night blindness type 2; PCA, principal component analysis; FDR, false discovery rate.

**Fig. 5:**
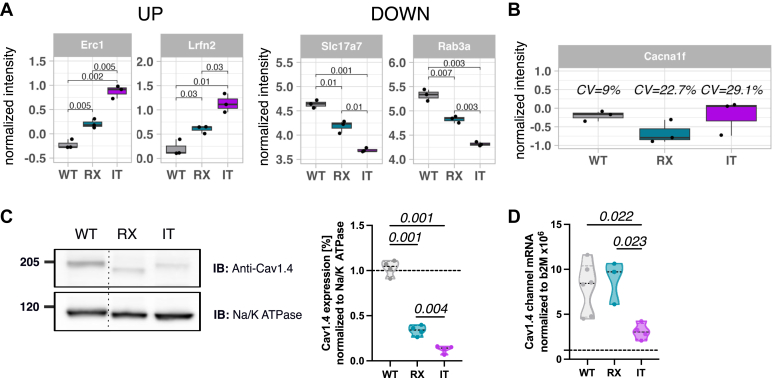
**Marker protein levels and nRNA quantification.***A*, protein abundances of membrane associated photoreceptor marker proteins (Erc1 (Q99MI1), Lrfn2 (Q80TG9), Slc17a7 (Q3TXX4), and Rab3a (P63011)) were plotted as normalized intensity by median subtraction (*y*-axis) across all mouse models. Statistical analyses with two-tailed unpaired *t* test. N = 9; n = 3 for all pooled samples (WT, RX, and IT). *B*, protein abundance of Cav1.4 including CV. *C*, representative western blots of protein levels of WT (N = 4), RX (N = 4), and IT (N = 4) retinas (each point represents a pool of three mice). The α1 signal was normalized to the sodium-potassium pump. The levels of Cav1.4 channels were decreased in RX (0.34 ± 0.03, N = 4) and IT (0.13 ± 0.02, N = 4) retinas compared to wild type retinas (1.03 ± 0.04, N = 4). *D*, absolute mRNA quantification of WT (N = 6), RX (N = 3) and IT (N = 4) normalized to the housekeeping gene b2M. Statistical analysis: One-way Anova with Tukey *post hoc* test; ns: *p* > 0.05; *p* value is indicated above; only significant *p* values are stated. CV, coefficient of variation.

**Fig. 6:**
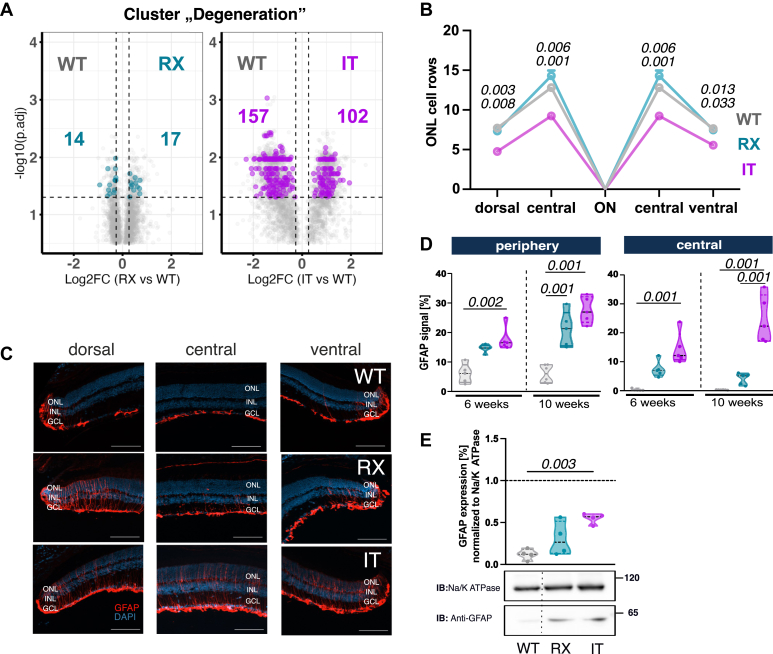
**Investigation of retinal stress markers in IT and RX.***A*, volcano plots (taken from Fig. 4*C*) with highlighted dysregulated proteins from the degeneration clusters in the IT, numbers indicate significantly dysregulated protein abundances. *B*, in the spider plot, the numbers of ONL rows of RX and IT were compared to wild type in the center and periphery of dorsal or ventral retinas. WT: dorsal: 7.72 ± 0.15; ventral: 7.64 ± 0.35; central: 12.8 ± 0.22; N = 5; RX: dorsal: 7.3 ± 0.48; ventral: 7.47 ± 0.37; central: 14.3 ± 0.66; N = 6; IT: dorsal: 4.76 ± 0.43; ventral: 5.56 ± 0.42; central: 9.24 ± 0.66; N = 5. *p* value is indicated above; *top*: WT versus IT; *bottom*: RX versus IT. *C*, GFAP localization in wild type, RX, and IT retinae. Sections were labeled with antibodies against GFAP (*red*) as a marker for reactive Müller cells and counterstained with DAPI (*blue*). WT: 6 weeks (N = 5) 10 weeks (N = 6); RX: 6 weeks (N = 5) 10 weeks (N = 5); IT: 6 weeks (N = 5) 10 weeks (N = 6); the scale bar represents 100 μm. *D*, overall GFAP signal in 6- and 10-week old mice. *E*, relative GFAP levels in mutant mice compared to wild type. WT: N = 4, HA = 4, RX = 4, IT = 4. Statistical analysis: One-way Anova with Tukey *post hoc* test; ns: *p* > 0.05; *p* value is indicated above; only significant *p* values are stated. DAPI, 4′,6-diamidino-2-phenylindole; GFAP, glial fibrillary acidic protein; ONL, outer nuclear layer.

### Effects of Pathogenic Cav1.4 Variants on the Remodeling of Second-Order Neurons

Disruption of synaptic transmission caused by pathogenic CSNB2 variants can lead to neuronal sprouting and reorganization of the retina (6, 10, 13, 28, 29, 30). This compensatory process involves the formation of new connections by secondary neurons, including bipolar and HCs, in response to impaired signaling between photoreceptors and their targets (31).

Our examination of RX retinas also revealed neurite sprouting of second order neurons (Fig. 3*A*), particularly in RBCs. Quantification of ONL occupancy revealed that the extend of rod bipolar dendritic sprouting in the RX variant was similar to that observed in the IT variant (Fig. 3*B*). Notably, CBCs seemed to be largely unaffected by the RX variant, exhibiting only few sprouts and therefore significantly lower dendritic occupancy compared to those in the IT variant (Fig. 3, *A* and *B*). Also, HC processes in RX retinas extended in the ONL (Fig. 3*C*). To distinguish between dendritic and axonal processes, we used NF200 and calbindin markers. Double-labeled processes indicated axons contacting rods, while single-labeled (calbindin only) processes represented dendrites connecting to cone terminals (32). Our analysis of RX retinas revealed that most sprouting processes (approximately 90%) were double-labeled, indicating that they were predominantly axonal (Fig. 3, *C* and *D*). In contrast, HC dendrites maintained their position within the OPL (Fig. 3*C* merge, Calbindin in magenta). A similar pattern of axonal rewiring was also observed in the IT variant (Fig. 6, (10)). However, when comparing the occupancy of calbindin labeled cells, we observed a lower occupancy in RX compared to IT retinas (Fig. 3*E*). This observation reflects the preservation of HC dendrites and their connections with cone terminals in the OPL of RX retinas. The minimal impact on CBCs suggests that the RX variant causes less severe structural disruption than the IT variant.

### Proteomic Characterization of Dysregulated Proteins in Retina Samples

To investigate how CSNB2 variants can influence retinal protein expression patterns, we developed a tailored proteomics technique to detect and quantify protein imbalances at the photoreceptor ribbon synapse. The workflow involves two centrifugation steps after cell lysis followed by in gel digest, similar to the approach for inner hair cells (33). We used the DIA mode, which systematically fragments all ions within predefined mass windows. This workflow allowed us to identify approximately 4.000 retinal protein groups per sample (Supplemental Fig. S2*A*) including low-abundant membrane proteins, such as Cnga3 (Q9JJZ8) and Lrit1 (Q8K099), while substantially reducing the number of animals needed to achieve reproducible results, requiring only three mice.

When we measured multiple proteins between different groups, we found that most proteins showed reproducible levels with little variation, and only a few proteins were more variable in their measurements than the others, as reflected by the tails of the violin plots (see coefficient of variation per group in Supplemental Fig. S2*B*, top). This suggests the experimental method was generally reliable. Among all groups in the synapse enriched preparation the IT samples showed the highest mean variability (15.6%), followed by WT (12.8%), whereas RX samples had the lowest variability (10.7%). This indicates that the quantitative measurements were more reproducible between replicates in the RX condition. These findings contrast with the whole retina approach (Supplemental Fig. S2*B*, bottom) where the CV values were clustered around approximately 20% in all groups.

To confirm the reliability of our dataset, we searched for key markers associated with rods and cone photoreceptor membranes (Fig. 4*A*). Our investigation focused on the following proteins: Prph2 (P15499), Cnga1 (P29974), Rom1 (P32958), Gnat1 (P20612), Cnga3 (Q9JJZ8), Grk1 (Q9WVL4), Opn1sw (O35599), and Rho (P15409). Thus, the analysis successfully identified integral and peripheral membrane protein markers localized in both rod and cone photoreceptors, validating our dataset's comprehensiveness. We consistently observed reduced levels of all rod protein markers in both CNSB2 variants (Fig. 4*A*). However, the IT variant also exhibited a more pronounced decrease in cone-specific markers, such as Cnga3 and Opn1sw (Fig. 4*A*). The IT variant exhibited broader downregulation that correlated with compromised cone photoreceptor function, a phenotype that was absent in the RX variant. Instead, the effects in the RX variant were limited to rod photoreceptors, as also evidenced by the rod-specific functional deficits (Fig. 1, *B* and *C*) and morphological changes (Fig. 3, *A* and *B*).

Our first step involved an unsupervised multivariate statistical technique, as such PCA, on the protein groups that had been quantified. The first principal component (PC1) accounted for 80.6% of the variance, with the second component (PC2) explaining 5.7%, highlighting clear clustering patterns among the samples (Fig. 4*B*). Wildtype and RX variant clusters were positioned in a close proximity, indicating that the RX variant causes only subtle proteomic alterations while retaining similarities to the wildtype. In contrast, the IT variant cluster was clearly distinct, indicating a substantial shift in the proteome and suggesting profound molecular changes.

For further characterization, we first visualized our proteome dataset using volcano plots to define dysregulated proteins based on a fold-change (FC) threshold of at least 1.5 and an adjusted *p* value below 0.05 (*t* test, Benjamini–Hochberg correction) (Fig. 4, *C* and *E*). According to these criteria, we identified 86 dysregulated proteins (upregulated 59; downregulated: 27) in the RX variant cluster compared to the wild type (Fig. 4*C*, *left panel*). In contrast, the IT variant cluster exhibited in total 2203 dysregulated proteins (upregulated 993; downregulated: 1210) compared to wildtype (Fig. 4*C*, *right panel*). A Venn diagram revealed that the majority of dysregulated proteins in the RX variant were also observed in the IT variant (41 up and 22 down regulated proteins; Fig. 4*D*). This overlap suggests that both variants share common molecular alterations. However, the extensive dysregulation observed in the IT variant, where more than half of the proteins were found to be regulated, complicates the identification of specific pathways directly affected by the Cav1.4 mutation. Such widespread protein dysregulation might represent the downstream consequences of broad compensatory cellular adaptations that retinal cells implement to cope with the gain-of-function mutation. Notably, in IT, the most prominently depleted pathways, were related to metabolic processes (Supplemental Fig. S3, right panel) and pathways linked to regulation of synapse structure/activity, dendrite development, synaptic vesicle cycle, and vesicle-mediated transport in synapses (Supplemental Fig. S3, left panel).

Consequently, we focused our analysis on the RX variant, where protein regulation appeared more selective and potentially linked to the direct consequences of the Cav1.4 mutation in CSNB2 pathogenesis. GO pathway analysis of the RX variant e.g. revealed enriched pathways related to postsynaptic organization, calcium ion transport, and protein localization to cell periphery (Fig. 4*E*, top panel). Moreover, the RX variant, showed depletion of pathways associated with visual perception, detection of light stimulus and sensory perception of light stimulus (Fig. 4*E*, bottom panel).

### Changes in the Protein Composition in Enriched Synapse Preparations

We subsequently summarized the GO results via keyword-based clustering (keywords are described in method section) to obtain a broader overview of dysregulated pathways. Given the low CV in our proteome data (Supplemental Fig. S2*B*), we now applied a more permissive FC threshold of 1.2. In the RX variant, we found enrichment for pathways related to “degeneration”, “metabolism” and “calcium” (Supplemental Fig. S4*A*). Downregulated pathways included synapse-related processes (Cluster “Synapse”), such as synaptic vesicle maturation, synaptic vesicle cycle, and vesicle-mediated transport in synapses. (Supplemental Table S2 *“*combined_clusters_ proteins_keywords_1.2”).

To further refine these findings, we extracted proteins from the keyword clusters (“Synapse“, “Vision“, “Calcium“) and analyzed them using STRING (Supplemental Fig. S4*B*). This revealed dysregulation of several key synaptic proteins, including critical components for the ribbon active zone. For example, in the “Synapse” cluster (Supplemental Fig. S4*B*), the calcium signaling protein Erc1 (Q99MI1, (34, 35, 36)) and the cone specific Leucine Rich Repeat and Fibronectin Type III Domain Containing 2 protein (Lrfn2/Q80TG9 (37)) were elevated, whereas vesicle associated proteins, including Rab3a (P63011, (38)) and vGlut1 (Slc17a7, Q3TXX4, (39)) were reduced (Fig. 5*A*). In the “Calcium” cluster, we also observed elevated α2δ subunits, with α2δ3 showing significant upregulation in RX (FC = 1.5). Given its role in promoting calcium channel trafficking to the membrane (40), this increase may represent a compensatory adjustment (Supplemental Fig. S4*B*).

We further examined the abundance of Cav1.4 in our proteomic dataset. Peptides corresponding to Cav1.4 were detected, but they showed inconsistent abundances (Fig. 5*B* and Supplemental Fig. S5). The variability likely reflects both the low abundance and its membrane-associated nature and therefore prevented us from identifying statistically significant changes at the protein level. To confirm our observation, we conducted western blot analysis using membrane-enriched retinal lysates (Fig. 5*C*) which revealed a significant decrease in Cav1.4 channel levels: a 70% decrease in RX retinas and an even more pronounced 87% reduction in IT retinas compared to wildtype samples. To determine if transcriptional changes could account for this discrepancy, we quantified *Cacna1f* mRNA expression using qPCR techniques (Fig. 5*D*). RX retinas displayed mRNA levels similar to those of the wild type, suggesting no significant changes in transcription. Conversely, the IT variant showed decreased mRNA levels, aligning with previous findings (6).

### Effects on Retinal Stress and Degeneration

In the “Degeneration” cluster, RX retinas contained markedly few dysregulated proteins (31 total; 17 more abundant, 14 less abundant; compared to 259 total in IT), consistent with a rather mild stress response (Fig. 6*A* and Supplemental Table S3 “dysregulated_degeneration _proteins_1.2”). RX showed minimal changes in proteins associated with apoptosis and retinal stress pathways (Supplemental Fig. S6*A*, Supplemental Table S4 “dysregulated_apoptotic_proteins_1.2” and Supplemental Table S5 “dysregulated_stress_proteins _1.2”). Consistent with these findings, cell row counts in the ONL remained unchanged in RX retinas (Fig. 6B), while the IT variant exhibited a substantial decrease in nuclei across all regions compared to wildtype, corroborating also previous findings (6, 12)

Photoreceptor cell death is a well-established characteristic observed in mouse models of retinal diseases, such as the rd10 (Pde6b) mouse model (41), which shows persistent accumulation of glial fibrillary acidic protein (GFAP) in Müller glia cells (42). In wildtype retinas, GFAP localization is restricted to the ganglion cell layer, except in peripheral regions (Fig. 6*C*). Conversely, the RX variant showed enhanced GFAP signals throughout all cellular layers. At 6 weeks, quantitative analysis revealed similar GFAP occupancy levels in the peripheral regions for both CSNB2 variants, but only IT reached significance (RX: 14.7% ± 0.6; IT: 17.8% ± 1.8; N = 5; *p* value = 0.002). This effect is similar in central regions (RX: 7.2% ± 1.2; IT: 14.2% ± 2.4, *p* value < 0.001). At 10 weeks, peripheral GFAP levels increased in both variants (RX: 21.2% ± 2.7; IT: 27.6% ± 2.0; N = 5, *p* value < 0.001) while the central occupancy rose significantly only the IT variant (RX: 3.99% ± 0.9; IT: 24.2% ± 3.6; N = 5; *p* value < 0.001) (Fig. 6*D*). Western blot analysis confirmed that GFAP levels were significantly elevated in IT compared to WT but not in RX, indicating greater retinal stress in IT (Fig. 6*E*). Furthermore, RX whole-mounted retinas showed no sign of activated microglia; rather, the microglia exhibited a homeostatic state, marked by small cell bodies and elongated dendrites (Supplemental Fig. S6*B*). Together, our results demonstrated that RX retinas exhibit only mild signs of degeneration.

## Discussion

### Validation of Mouse Models for CSNB2 Research

The diverse clinical presentations of CSBN2 caused by Cav1.4 variants highlight the critical need for animal models that recapitulate human disease phenotypes when investigating underlying mechanisms. Recent comparative studies between mouse models and human patients demonstrate the importance of phenotypic fidelity when investigating underlying mechanisms.

Knoflach et al, (6) compared the functional phenotype of the IT variant in mouse with human, observing similar features in both. Their findings revealed abnormalities in rod- and cone-mediated flash responses, along with reduced scotopic a-wave amplitudes—an atypical presentation compared to the standard CSNB2 profile (8). This a-wave reduction contrasts with earlier reports defining CSNB2 by intact photoreceptor function (normal a-waves) paired with impaired bipolar cell transmission (diminished b-waves) (5).

The translational validity of mouse models is further reinforced by our studies of the RX variant. The patients carrying the RX variant exhibit the typical Schubert-Bornschein-type ERG pattern, consistent with CSNB2 (9, 14, 43). Importantly the RX mouse model also recapitulates this phenotype through reduced scotopic b-waves with intact a-waves, mirroring human electrophysiological findings. Moreover, our MEA recordings provided crucial validation and extension of these ERG findings. The combined results from both mouse models validate their utility for studying CSNB2 mechanisms.

### Variant-Specific Functional and Structural Phenotypes

Heterologous expression studies revealed distinct functional deficits between variants. RX channels exhibited activation at lower voltages than expected, characterized by a 10 mV negative shift in voltage sensitivity, and showed calcium-depended inactivation (14). In contrast, IT channels demonstrated a more pronounced hyperpolarizing shift (∼-30 mV) (7). These voltage shifts have important physiological implications. In darkness, photoreceptors experience continuous Ca^2+^ influx, which must be tightly regulated to prevent toxic overload (41). The substantial voltage shift in the IT variant likely leads to excessive calcium accumulation within photoreceptor terminals, exacerbating cellular stress. Although the RX variant also displays altered voltage sensitivity, the smaller shift (∼-10 mV) suggests less pronounced impacts on calcium homeostasis and subsequent signaling pathways.

To assess whether their structural characteristics corresponded to the functional differences observed in heterologous expression study and the ERG, we examined the morphology of our both mouse models. Previous studies have established the crucial role of Cav1.4 in photoreceptor development and maturation (6, 10, 11, 12, 13, 26, 29, 44, 45, 46, 47, 48). Our analysis revealed significant structural differences between variants. We (and others (6, 12)) observed a significantly thinner ONL layer in the IT variant, indicative of substantial photoreceptor degeneration—a feature not observed in the RX variant. Both RX and IT variants displayed reduced Cav1.4 protein levels, although the mechanisms likely differ. In IT retinas, the reduction in Cav1.4 channel abundance may in partially results from ONL thinning, which reduces the overall number of photoreceptor cells. This decrease aligns with the reduced Cav1.4 mRNA levels observed in IT.

#### Synaptic Ribbon Architecture and Active Zone Organization

Despite their distinct functional effects, both RX and IT variants exhibited similarly small synaptic ribbons. Previous research has demonstrated that Cav1.4 channels and ribeye are spatially correlated at the active zone, with ribbon length proportionally scaling with active zone size. Meaning that longer ribbons were always associated with larger active zones and, vice versa, smaller ribbons correspondingly with smaller active zones. These findings suggest that ribbons stabilize Cav1.4 and organizes nanodomain coupling of Cav-channels with release-ready vesicles, indicating that ribbon size influences active zone organization and nanodomain coupling (49).

Similarly, in inner hair cells ribbon size and presynaptic density directly correlates with the number of Ca^2+^ channels (50, 51, 52). However, an important consideration of our current analysis is that our ribbon length measurements do not distinguish between rods and cones. This is particularly relevant given that our molecular and functional data indicate effects primarily in rods, while the structural (ribbon length) findings appear similar across both photoreceptor types. Future studies should employ rod- and cone-specific labeling or imaging techniques to resolve this question and directly assess whether ribbon remodeling is selectively restricted to rods in RX variants.

#### Variant-Specific Molecular Pathway Responses

Under our experimental conditions, both variants exhibited comparable dysregulated pathways. However, the extent of dysregulation was markedly greater in the IT variant, as evidenced by a 20-fold increase in dysregulated proteins. Altered calcium homeostasis appears to be a key factor in triggering multiple apoptotic pathways (53), potentially involving both mitochondrial and endoplasmic reticulum-mediated mechanisms. In line with this, we detected a significant upregulation of several pro-apoptotic proteins in IT, most notably mammalian target of rapamycin, a key regulator of stress signaling at mitochondria-associated endoplasmic reticulum membranes. Notably, these apoptotic markers and stress-related pathway dysregulations were absent in RX retinas, further underscoring the milder, nondegenerative phenotype associated with this variant.

Although the RX variant also induced alterations in synaptic and calcium-related pathways, these changes were considerably milder and did not extend to apoptotic signaling. Our proteomic clustering analysis revealed a potential mechanism in calcium handling in the RX, by the "Calcium" cluster. RX retinas show upregulation of calcium ion transport and homeostasis pathway. These findings suggest a response to intracellular calcium overload in the RX variant, likely caused by the left-shift of channel activation. However, this mechanism appears insufficient and persistently elevated basal calcium levels in RX retinas might lead to excessive calcium-dependent exocytosis (54, 55, 56). This dysregulation could ultimately deplete the available vesicle pool, leading to impaired neurotransmission (57, 58).

In support of this hypothesis in the RX variant showed upregulation of the active zone protein Erc1 (Fig. 5A), an important scaffolding protein involved in mediating calcium influx and anchoring components of the secretory machinery at the active zone (36, 59). Increased Erc1 abundance as a critical scaffolding protein could improve vesicle tethering and increase the size of the readily releasable pool (60). The RX variant also showed a reduction in synaptic vesicle-associated proteins, specifically, a downregulation of Rab3a and vGlut1 (encoded by the gene Slc17a7). vGlut1 is essential for loading glutamate into the synaptic vesicles and ensuring proper recycling for efficient synaptic transmission (61). Thus, its downregulation may therefore cause an impairment in vesicle filling and neurotransmission efficiency.

These results align with data from Ribeye-deficient animal models, where active zone proteins were reduced (49) and fewer vesicles were associated with the membrane, ultimately impairing neurotransmitter release—primarily due to an impaired ribbon structure (25).

Together these data suggest that RX variants undergo synaptic remodeling. Although increased vesicle turnover may temporarily support neurotransmission, it could eventually result in synaptic exhaustion. Over time, this imbalance would drive progressive photoreceptor dysfunction and retinal degeneration.

#### Differential Photoreceptor-Specific Vulnerability to Cav1.4 Dysfunction

The differential effects of *CACNA1F* mutations on rod and cone pathways reveal photoreceptor-specific synaptic mechanisms, where Cav1.4 dysfunction leads to more nuanced phenotypes. For example, the nonconducting G369i variant showed a loss of rod-derived signaling, while preserving cone function (26, 62). In contrast, the nob2 mouse model—where Cav1.4 protein levels are reduced but some calcium channel function is still retained (63)—showed a reduced but not absent b-wave under scotopic conditions (29).

Of note, RX and IT variants also exhibited reduced Cav1.4 protein levels, as shown by western blot (Fig. 5*C*), but the underlying mechanisms may differ. In the IT variant, reduction of Cav1.4 also resulted from ONL thinning (Fig. 6*B*). Still, a parallel can be drawn to a recent study, describing a Cav1.3 gain-of-function mutation (A749G; AG) that caused a ∼20 mV hyperpolarizing shift in voltage sensitivity (64, 65). The increased channel activity led to partial loss of synaptic ribbons likely through Ca^2+^-dependent mitochondrial signaling, and channel abundance was reduced as a result of this ribbon reduction (64). However, this mechanism has only been investigated directly in inner hair cells (66). Although studies in photoreceptors have established that increased mitochondrial Ca^2+^ uptake (e.g., via mitochondrial calcium uniporter overexpression) leads to altered metabolism and accelerated photoresponse recovery (67), these experiments did not examine synaptic ribbon structure or Ca^2+^ channel abundance. Thus, there is currently no direct evidence for the same pathway in photoreceptor ribbon synapses. Nevertheless, CSNB2 models show similar patterns: both RX and IT variants have leftward shifts in activation (RX: −10 mV; IT: −30 mV) and reduced Cav1.4 protein levels (Fig. 5*C*). This suggests that altered gating can trigger synaptic remodeling that ultimately reduces channel abundance in both Cav1.3 and Cav1.4. Nevertheless, this mechanism cannot explain why RX specifically affects rods.

The selective vulnerability between photoreceptor types raises an important question: what makes cones more resilient to Cav1.4 disruption? Cone-specific proteins may help preserve synaptic function in cones despite Cav1.4 dysregulation. One such example is Lrfn2, which is exclusively localized in cone photoreceptors (37) and upregulated in IT and RX mice in our study (Fig. 5*A*). Interestingly, knocking out of Lrfn2 did not change presynaptic protein levels of Elfn2 and Lrit3 which are other trans-synaptic scaffolding proteins that are important for structural stability and correct synaptic wiring with postsynaptic cone partners. However, functional analysis (ERG) revealed a reduction in the photopic b-wave amplitude, indicating impaired cone-mediated transmission. Lrfn2 is localized at the cone pedicle base (37, 68), where it specifically connects with OFF bipolar cells (68); other work suggested a connection to ON bipolar cells (37). Soto et al, recently confirmed that Lrfn2 is required for signal transmission from cones to OFF bipolar cells (68). However, the exact mechanism how Lrfn2 exerts trans-synaptic control still remains elusive. Previous research in the brain has shown that Lrfn2 contributes to synaptic plasticity and synapse development (69). Although AMPA receptor-mediated currents were reduced in the brain, excitatory synapse density, size, and morphology were only minimally altered (69). Based on these findings, it is plausible that Lrfn2 in cones similarly contributes to organize and stabilizing postsynaptic components, such as Trpm1, GluK1, and GluA1 (68), potentially helping to maintain bipolar cell responses under conditions of altered glutamate release, however not dysregulated in our CSNB2 mouse models.

#### Proteomic Validation and Technical Considerations

Previous proteomic studies have provided valuable insights into the retina’s global protein composition and its alterations in various conditions (70, 71, 72, 73) but detecting low abundance proteins, especially synaptic proteins, remains challenging. To address this limitation, we employed a proteomic approach based on DIA (74, 75) which significantly enhances proteome coverage - as demonstrated by Montaser et al (76), who reported a more than four-fold improvement in overall proteome coverage.

To our knowledge, this study represents the first comprehensive proteome analysis to successfully detect low-abundance synaptic proteins in the retina, including voltage-gated calcium channels. In addition to calcium channels our approach allowed us to successfully identify membrane-associated proteins of rod photoreceptors and low-abundance cone photoreceptor proteins, like Cnga3, Lrit1, and SV2A. These proteins are known to be present at significantly lower levels compared to highly abundant retinal proteins like rhodopsin or housekeeping proteins such as GAPDH, often showing a 1000-fold difference in abundance.

This study's findings can be contrasted with two recent proteomic analyses: the first by Lux et al, used fluorescence activated cell sorting to isolate rods and cones (77). While also innovative, their approach did not detect low-abundance calcium channels, possibly due to insufficient enrichment of synaptic proteins. Similarly, Cepeda *et al*. (33) conducted a proteome analysis of inner hair cells focusing on ribbon synapse proteins under native conditions using a similar enrichment technique than ours. Despite this targeted approach, they were unable to detect the Cav1.3 calcium channel, which is crucial for ribbon synapses in inner hair cells (78, 79, 80). Still, a major constraint in our study is exact subcellular localization of the presynaptic proteins. We cannot entirely rule out the possibility that some of these proteins may be present in other retinal synapses beyond the photoreceptor synapse. Nevertheless, we have mitigated this potential limitation by choosing isoforms that are known to be primarily localized at photoreceptor synapses.

PCA demonstrated a high degree of experimental reproducibility, though we noted a certain degree of variability within the IT variant. This heterogeneity likely stems from the ONL cell loss that disrupts the normal retinal layer composition compared to wild-type specimens (76). Therefore, bulk retinal proteomics in degenerative samples must be interpreted with caution due to shifts in retinal layer proportions. As photoreceptors degenerate, the relative proportion of other retinal regions increases, leading to an apparent loss of photoreceptor proteins and a relative enrichment of inner retinal proteins, even if their per-cell expression remains unchanged (76). This heterogeneity underscores the importance of considering structural changes and/or disease progression when interpreting proteomic data from retinal disease models, as shifts in retinal layer composition can influence the abundance of specific proteins.

## Data Availability

All data generated or analyzed during this study are included in this published article (and its supporting information files). The mass spectrometry proteomics data have been deposited to the ProteomeXchange Consortium (http://prot eomecentral.proteomexchange.org) via the PRIDE (81) partner repository with the dataset identifier PXD063923.

## Supplemental data

This article contains supplemental data.

## Conflict of interest

The authors declare no competing interests.
