# Supplementary material for: Quantitative Proteomics Identifies Potential Molecular Adaptations in Mouse Models of Congenital Stationary Night Blindness Type 2
Source: Mol Cell Proteomics. 2025 Nov 10;24(12):101462. doi: 10.1016/j.mcpro.2025.101462 (PMC12802112; doi:10.1016/j.mcpro.2025.101462)
Supplement: Supplemental Data [file mmc1.pdf]

Supplemental Data for

Quantitative proteomics identifies potential molecular adaptations in mouse models of congenital stationary night blindness type 2

Matthias Ganglberger[1], Lucia Zanetti[1], Anna-Sophia Egger[2], Alexander Günter[3], Bettina Wagner[4], Soumaya Belhadj[3], Regine Mühlfriedel[3], Dagmar Knoflach[5], Emilio Casanova[5,6], Thomas Rülicke[4], Mathias W. Seeliger[3], Marcel Kwiatkowski[2], Hartwig Seitter[1] and Alexandra Koschak[1*]

* Correspondence:

Alexandra Koschak, University of Innsbruck, Institute of Pharmacy, Pharmacology and Toxicology, Center for Chemistry and Biomedicine, Innrain 80-82, A-6020 Innsbruck, Austria, phone: +43-(0)512-507-58807, Fax: +43-(0)512-507-58899, email: alexandra.koschak@uibk.ac.at.

Table of Contents

Supplementary Figure 1. Targeting Strategy, Protein Expression, and Retinal Activity Analysis of the Cav1.4-RX Mouse Model S-2

Supplementary Figure 2. Comparison of Protein Quantification and Normalization Across Synapse-Enriched and Whole Retina Samples S-4

Supplementary figure 3: GO enrichment analysis of dysregulated proteins in the IT variant, with a fold change of 1.5. .... S-5

Supplementary figure 4 GO Pathway Clustering and Protein Interaction Analysis in the RX Variant S-6

Supplementary figure 5. Boxplot of Peptide abundances of the Cav1.4 proteins (Mean ± SD)......... S-7

Supplementary figure 6. Analysis of Degeneration-Associated Proteins and Retinal Microglia Morphology in RX and WT Mice S-8

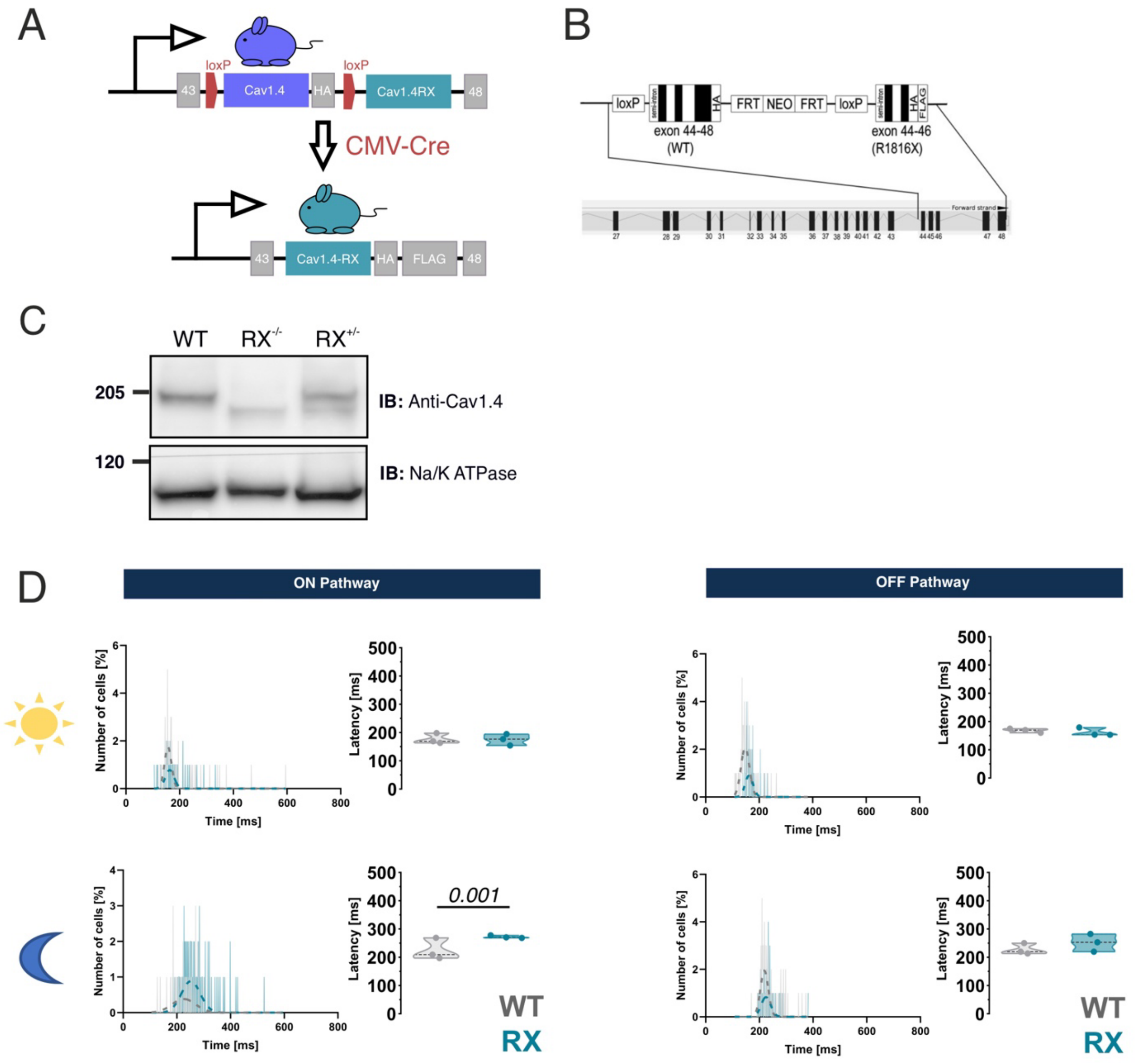

Supplementary Figure 1. Targeting Strategy, Protein Expression, and Retinal Activity Analysis of the Cav1.4-RX Mouse Model

A: General structure of the targeting. The Cav1.4-WT full length channel is flanked by loxP sited and labelled with a HA-tag. The truncated channel RX is marked with a HA/FLAG-tag. B: The final targeting construct. The transcription of both constructs, Cav1.4-HA and RX, starts with a semi-intron. For the wildtype channel the exons 44-48 were extracted from cDNA. These 5 exons replaced the exons 44 – 48 within the genomic DNA and the final WT construct was flanked by loxP sites. The FRT-NEO-FRT cassette expresses the neomycin resistance gene which will be used for selection of transfected embryonic stem cells. The targeting construct Cav1.4-RXHA/FLAG consists of exon 44-46 and were also extracted from Cav1.4 cDNA. By crossing transgenic mouse with cre mouse line, the RX protein is synthesized. C: Representative western blot of WT and hemi- and heterozygous RX retinas. Loading control: sodium-potassium pump. D: Ex vivo retinal activity. MEA were performed from retinal whole-mounts. Full-field flash (FFF) stimulus consisting of negative contrast (dark flash) and positive contrast (bright flash) 50% Weber contrast; bottom: response in scotopic luminance (moon) and top: photopic luminance (sun).

Traces show average responses of WT (grey, N = 3) and RX (green, N = 3). Mean latencies of OFF and ON ganglion cells under scotopic and photopic conditions. Data points represent all the ganglion cells analysed, WT: n scotopic = 42, n photopic = 36; RX: n scotopic = 91, photopic = 89, while the violin plot represent the mean latency per animal in WT (in [ms]: scotopic: 227.8 ± 11.27; photopic: 168.2 ± 4.20 , N = 3) and RX (in [ms]: scotopic: 251.9 ± 18.14; photopic: 161.8 ± 8.53, N = 3). left panel: mean latencies of ON GC responses in WT (in [ms]: scotopic: 225.2 ± 22.07 n= 42; photopic: 176.9 ± 10.73, n= 43, N = 3) and RX (in [ms]: scotopic: 272.4± 2.77, n = 87 ; photopic: 175.4 ± 11.72, n = 74, N = 3). right panel: mean latencies of OFF GC responses in WT (in [ms]: scotopic: 227.8 ± 11.27; photopic: 168.2 ± 4.20 , N = 3) and RX (in [ms]: scotopic: 251.9 ± 18.14; photopic: 161.8 ± 8.53, N = 3). Statistics: Kruskal-Wallis test with Dunn's Multiple Comparison Test. p value is indicated above.

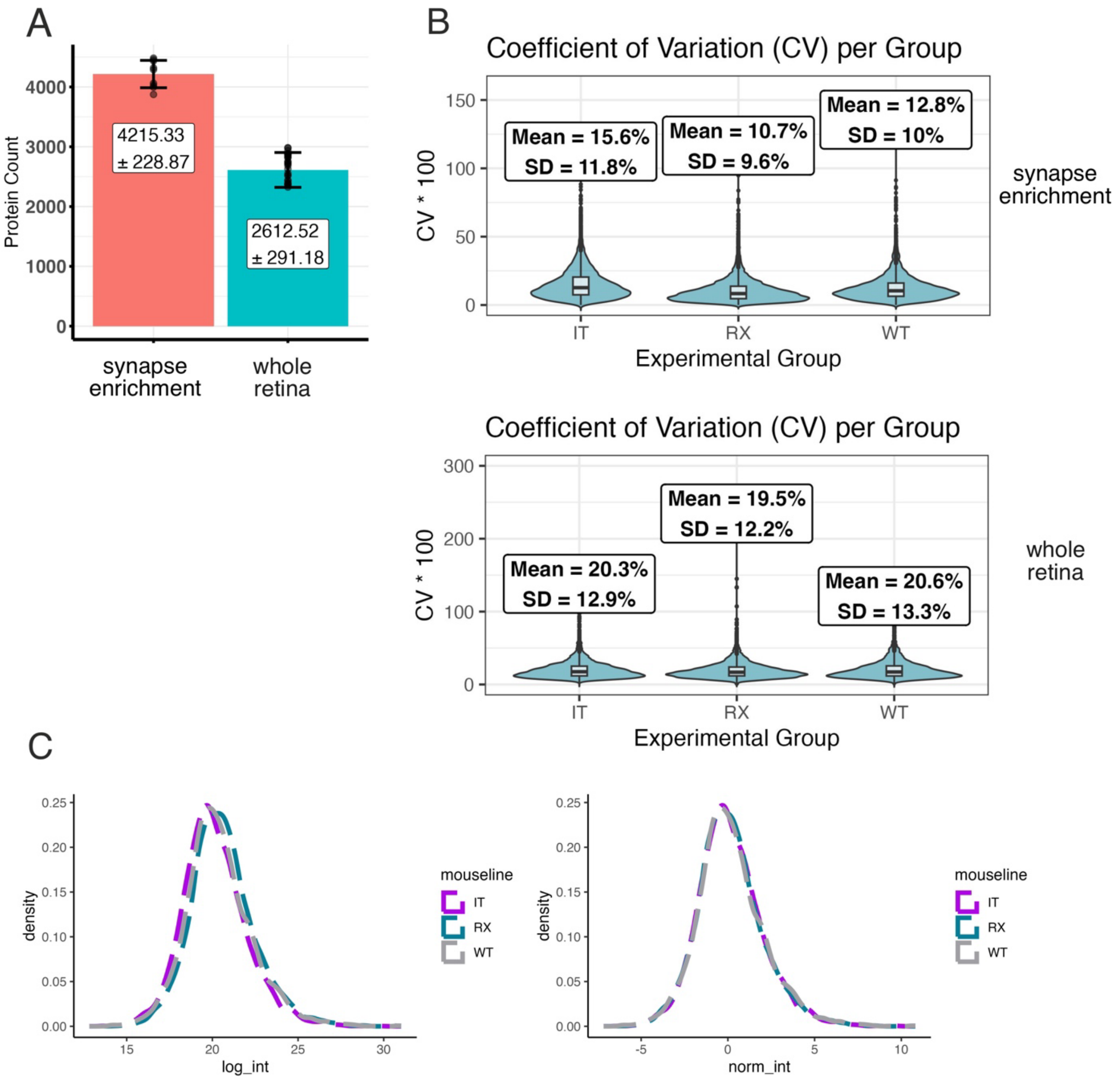

Supplementary Figure 2. Comparison of Protein Quantification and Normalization Across Synapse-Enriched and Whole Retina Samples

A: Bargraph of identified protein groups with different conditions. Synapse enriched samples: n = 9; whole retina samples n = 29; (Mean ± SD). B: Coefficient of variation (CV) of the synapse enriched (top panel) and the whole retina approach (bottom panel) (Mean ± SD). C: Density plots of log2-transformed protein intensities for each experimental group (IT, RX, WT). The left panel shows raw intensities prior to normalization, while the right panel shows intensities after median normalization within each condition. Normalization effectively centers the distributions, ensuring comparability across groups

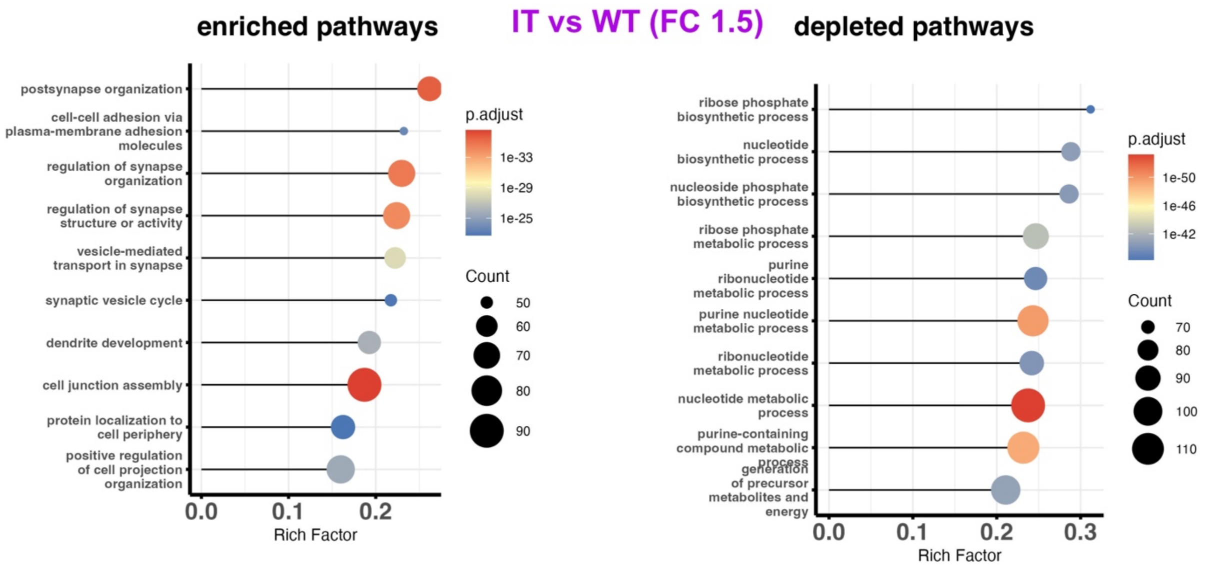

Supplementary figure 3: GO enrichment analysis of dysregulated proteins in the IT variant, with a fold change of 1.5.

The dot plot presents the Rich factor (x-axis) against the number of proteins (y-axis), with adjusted p-values (p.adjust) represented by color coding.

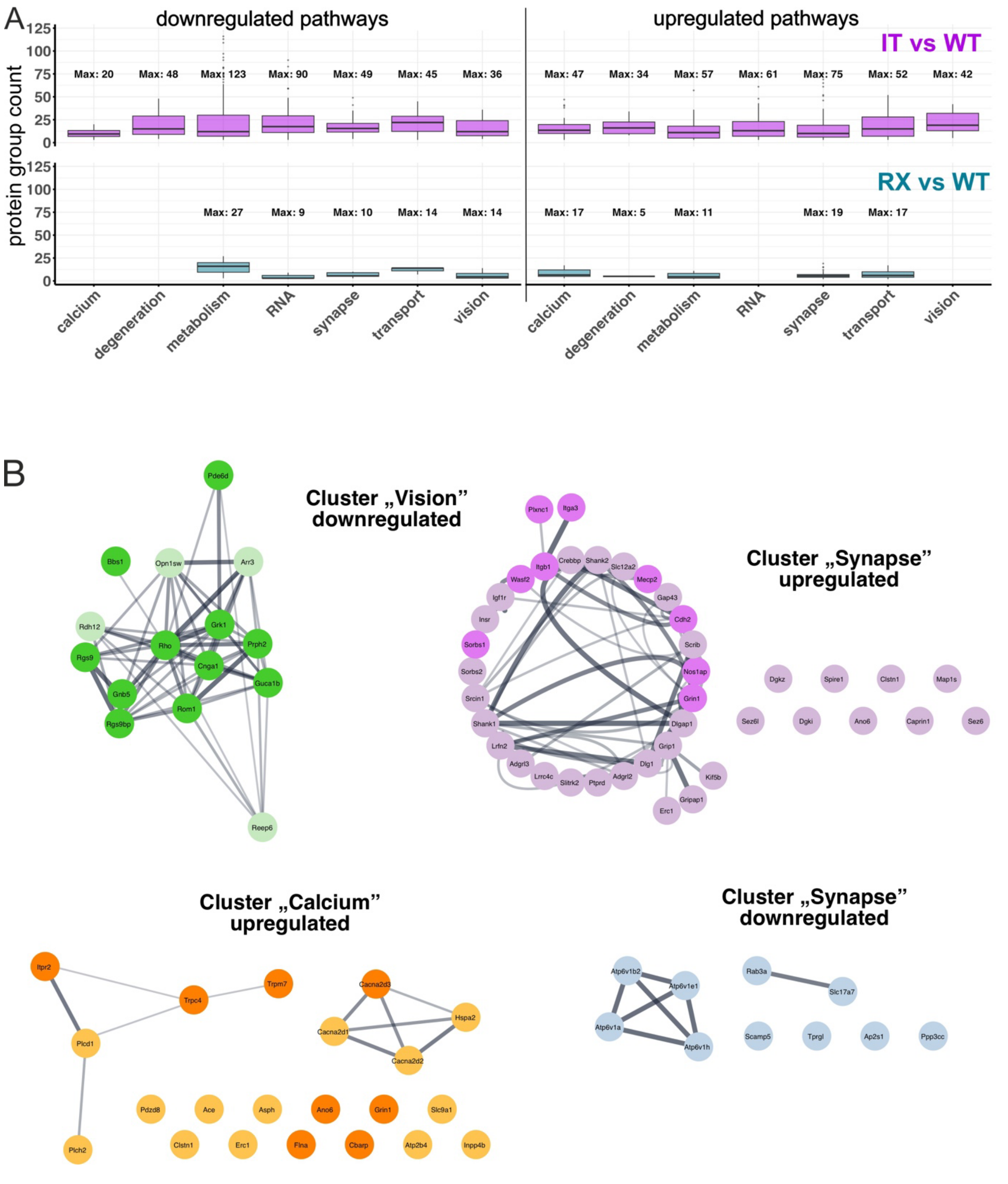

Supplementary figure 4 GO Pathway Clustering and Protein Interaction Analysis in the RX Variant

A: GO pathways clustered by selected keywords with a fold change ≥ 1.2. Numbers indicate the mean of all proteins referred in respective cluster. B: Visualization of selected proteins associated with the clusters shown in panel A from the RX variant, using interaction data from the STRING database. Edge thickness reflects the STRING interaction score. Darker-colored nodes indicate proteins with a fold change ≥ 1.5, whereas lighter-colored nodes represent proteins with a fold change ≥ 1.2.

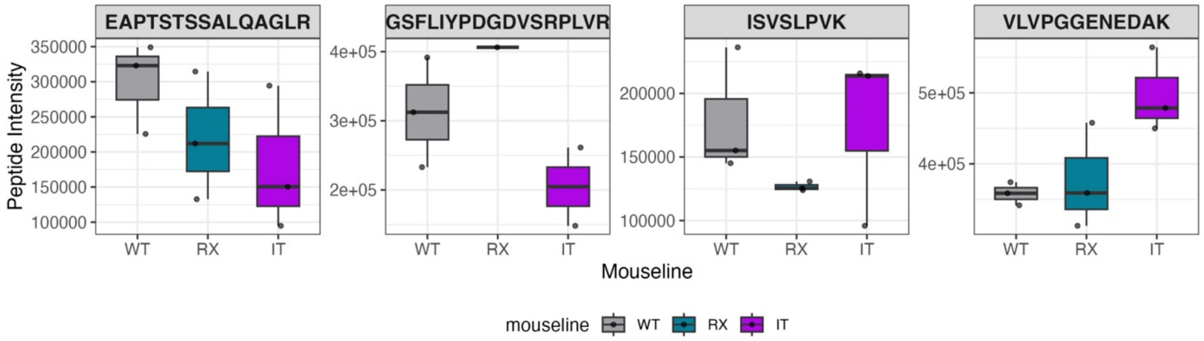

Supplementary figure 5. Boxplot of Peptide abundances of the Cav1.4 proteins (Mean ± SD).

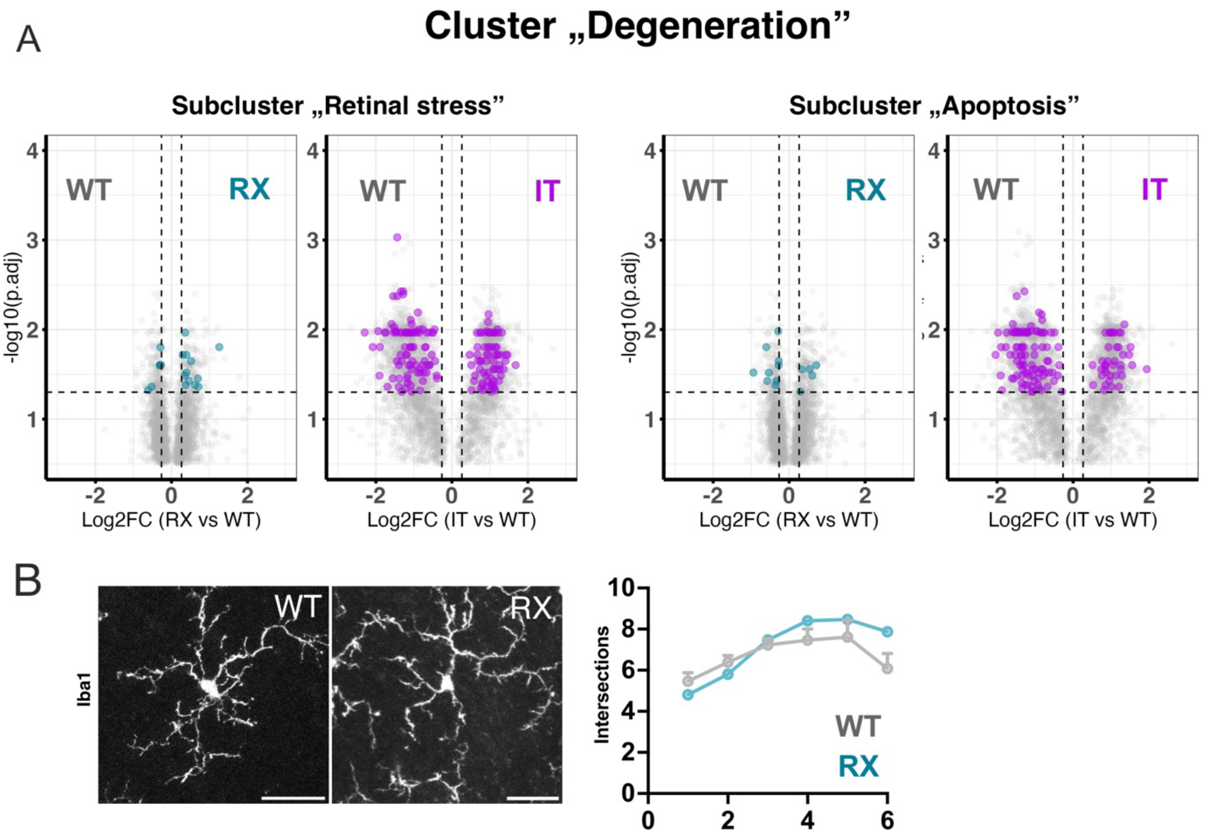

Supplementary figure 6. Analysis of Degeneration-Associated Proteins and Retinal Microglia Morphology in RX and WT Mice

A: Vulcano plots (taken from Figure 4C) with highlighted dysregulated proteins from the subclusters of "Degeneration". B: Representative retinal microglia in adult mice (10 weeks) from RX (N = 3) and WT mice (N = 3) were stained with microglia marker Iba1. Scale bar = 10 µm. Sholl analysis of RX (n = 15; N = 3) and WT (n = 13; N = 3) microglia.
